# Alterations in regional homogeneity of resting-state cerebral activity in patients with chronic prostatitis/chronic pelvic pain syndrome

**DOI:** 10.1371/journal.pone.0184896

**Published:** 2017-09-19

**Authors:** Yusong Lin, Yan Bai, Peng Liu, Xuejuan Yang, Wei Qin, Jianqin Gu, Degang Ding, Jie Tian, Meiyun Wang

**Affiliations:** 1 The Cooperative Innovation Center of Internet Healthcare & School of Software and Applied Technology, Zhengzhou University, Zhengzhou, China; 2 Department of Radiology, Zhengzhou University People’s Hospital & Henan Provincial People's Hospital, Zhengzhou, China; 3 Henan Key Laboratory for Medical Imaging of Neurological Disease, China; 4 Medical School, Henan University, Zhengzhou, China; 5 School of Life Sciences and Technology, Xidian University, Xian, China; 6 Henan Provincial Clinical Big Data Analysis and Service Engineering Research Center, Zhengzhou University People’s Hospital and Henan Provincial People’s Hospital, Zhengzhou, China; 7 Department of Urology, Zhengzhou University People’s Hospital & Henan Provincial People's Hospital, Zhengzhou, China; 8 Institute of Automation, Chinese Academy of Sciences, Beijing, China; Institute of Psychology, Chinese Academy of Sciences, CHINA

## Abstract

The purpose of this study was to explore the neural mechanism in Chronic prostatitis/Chronic pelvic pain syndrome (CP/CPPS) using resting-state functional magnetic resonance imaging. The functional magnetic resonance imaging was performed on 31 male CP/CPPS-patients and 31 age and education matched male healthy controls on a 3-T magnetic resonance imaging unit. A two-sample t-test was adopted to reveal the regional homogeneity between the patients and healthy controls. The mean regional homogeneity values in the alerted brain regions of patients were correlated with the clinical measurements by using Pearson's correlation analyses. The CP/CPPS-patients had significantly decreased regional homogeneity in the bilateral anterior cingulate cortices, insular cortices and right medial prefrontal cortex, while significantly increased regional homogeneity in the brainstem and right thalamus compared with the healthy controls. In the CP/CPPS-patients, the mean regional homogeneity value in the left anterior cingulate cortex, bilateral insular cortices and brainstem were respectively correlated with the National Institutes of Health Chronic Prostatitis Symptom Index total score and pain subscale. These brain regions are important in the pain modulation process. Therefore, an impaired pain modulatory system, either by decreased descending pain inhibition or enhanced pain facilitation, may explain the pain symptoms in CP/CPPS.

## Introduction

Chronic prostatitis/Chronic pelvic pain syndrome (CP/CPPS), the most frequent form prostatitis in men [1], has a prevalence rate range from 9% to 16% in the world [2]. Chronic genitourinary pain is more impact on the quality of life than other CP/CPPS symptoms [3]. Unfortunately, the pathogenesis of CP/CPPS has been largely unknown so far [4]. The patients with CP/CPPS always have consultations with many doctors and a long history of suffering [5]. Thus, CP/CPPS imposes a substantial economic cost on the individual and society [5]. The local pelvic inflammation may be one of the pathophysiological correlates in some patients with CP/CPPS [6]. The peripheral sensitization caused by the inflammation factors may underlie the chronic pain in the patients with CP/CPPS. In addition, CP/CPPS is usually associated with the pelvic discomfort, negative cognitive, sexual, behavioral or emotional consequences [7], which may influence the sensation and function of the brain. But the chronic pain is the characteristic symptom of CP/CPPS [5]. The brain has been proved to be involved in the pain modulation of CP/CPPS [8,9]. The previous studies have reported that the anterior cingulate cortex (ACC) and insular cortex (INC) were altered in the CP/CPPS-patients [8,9]. Until recently, only few studies have used functional or structural magnetic resonance imaging (MRI) to investigate the pain of CP/CPPS [8,9].

Regional homogeneity (ReHo) has been developed to explore the regional spontaneous activity with similar or synchronous hemodynamic characteristics in the voxelwise analysis [10]. ReHo was used to analyze the blood oxygen level-dependent signal in the brains of patients during the resting-state. Recently, ReHo has been extensively used to study cerebral alterations in patients with chronic pain [11].

In this study, we thereby applied the functional magnetic resonance imaging (fMRI) with ReHo method to investigate the neural mechanism of CP/CPPS in the patients without spontaneous pelvic pain during resting-state. We hypothesized that the altered ReHo would be found in the certain brain regions, especially related to the ACC and INC in CP/CPPS.

## Material and methods

### Patient population

31 male CP/CPPS-patients and 31 age and education matched male healthy controls were enrolled between October 2013 and May 2016 in this study. Both of the patients and healthy controls were right-handed. The inclusion criteria for CP/CPPS-patients were as follows: (a) the National Institutes of Health Chronic Prostatitis Symptom Index (NIH-CPSI) total score 15 or greater [12]; (b) patients without treatment for the CP/CPPS symptoms and (c) patients had complaints about pelvic pain at least 3 months within the last 6 months. We excluded 4 patients with bacterial infections in the prostates. Therefore, 27 patients (age range, 21–56 years; mean age ± standard deviation, 34.1 ± 10.3 years; education years range, 9–16 years; mean education years ± standard deviation, 10.7 ± 2.9 years) and 27 age and education matched healthy controls (age range, 21–56 years; mean age ± standard deviation, 34.1 ± 10.4 years; education years range, 9–16 years; mean education years ± standard deviation, 10.7 ± 2.8 years) were included for the analysis. The study size was arrived at a matched case-control study design. All patients during acquisitions of fMRI data were in the resting-states without spontaneous pelvic pain. This study was approved by the institutional review board of Zhengzhou University, and written informed consent was obtained from each subject before participation.

### Resting-state fMRI data acquisition

The resting-state fMRI data and high resolution T1-weighted magnetic resonance images were obtained by using a 3-T MRI unit (Discovery MR 750; General Electric Medical Systems, Milwaukee, Wisconsin) with an eight-channel phase array head coil. The fMRI images were acquired with an echo-planar imaging sequence (repetition time, 2000 milliseconds; echo time, 30 milliseconds; matrix, 64 × 64; field of view, 24 cm × 24 cm; slice thickness, 4 mm; no gap; 38 slices and total 210 time points). The high resolution T1-weighted magnetic resonance images were acquired with a three-dimensional fast spoiled gradient-echo dual-echo sequence (repetition time, 8.2 milliseconds; echo time, 3.2 milliseconds; matrix, 256 × 256; field of view, 24 cm × 24 cm; slice thickness, 1 mm; no gap and 156 slices). The authors had access to information that could identify individual participants during or after data collection in this case-control study.

### Resting-state fMRI data processing

The resting-state fMRI data were processed by the Statistical Parametric Mapping 5 (http://www.fil.ion.ucl.ac.uk/spm/software/spm5) and Data Processing Assistant for Resting-State Functional MRI V2.2 Basic Edition (http://www.restfmri.net) softwares. We discarded the first 10 volumes of each functional time series. The remaining images were corrected for acquisition time delay between different slices and realigned to the first volume. The head motion of each participant was less than 2 mm translation in any cardinal direction and 2 degrees rotation in x, y or z axe. The resting-state fMRI data were then spatially normalized to the Montreal Neurological Institute space. The linear drift and the average blood oxygen level-dependent signals in ventricular and white matter regions were removed. A band-pass filtration (0.01–0.08 Hz) was performed to reduce the noise. The Kendall coefficient of concordance was used to measure the correlation of the time series with its 26 nearest neighboring voxels. Individual ReHo maps were generated by calculating the Kendall coefficient of concordance. After the ReHo calculation, a Gaussian kernel with a full width at half-maximum of 4 mm was used to smooth the ReHo image in order to reduce noise and residual differences. The mean ReHo value of the voxels in the alerted brain regions was extracted for each patient.

### Behavioral measurements acquisition

NIH-CPSI total score, NIH-CPSI pain subscale, Zung Self-Rating Anxiety Scale (SAS) standard score [13] and Zung Self-Rating Depression Scale (SDS) standard score [14] were acquired from each participant before the MRI scan. The NIH-CPSI questionnaire was used to evaluate the CP/CPPS symptoms in the disease history. The NIH-CPSI pain subscale was related to the pain localization, pain symptom, pain frequency and pain severity. The visual analogue scale was used to confirm the patient state without spontaneous pelvic pain. The visual analogue scale test had to be 0 score within 10 minutes before and after the MRI scan, respectively.

### Statistical analysis

Two-sample t-tests were used to identify the differences in the whole brain ReHo maps between the CP/CPPS-patients and the healthy controls. Results were thresholded at p < 0.05 (false discovery rate corrected). The mean ReHo values in the alerted brain regions of patients were correlated with the NIH-CPSI total score and NIH-CPSI pain subscale by using Pearson's correlation analyses. Results were thresholded at p < 0.05/8 (Bonferroni corrected). The age, SAS standard score and SDS standard score were considered as covariances of no interest. The education was not included as a covariate due to its matching between the CP/CPPS-patients and the healthy controls.

## Results

### Resting-state fMRI data

**Fig 1:**
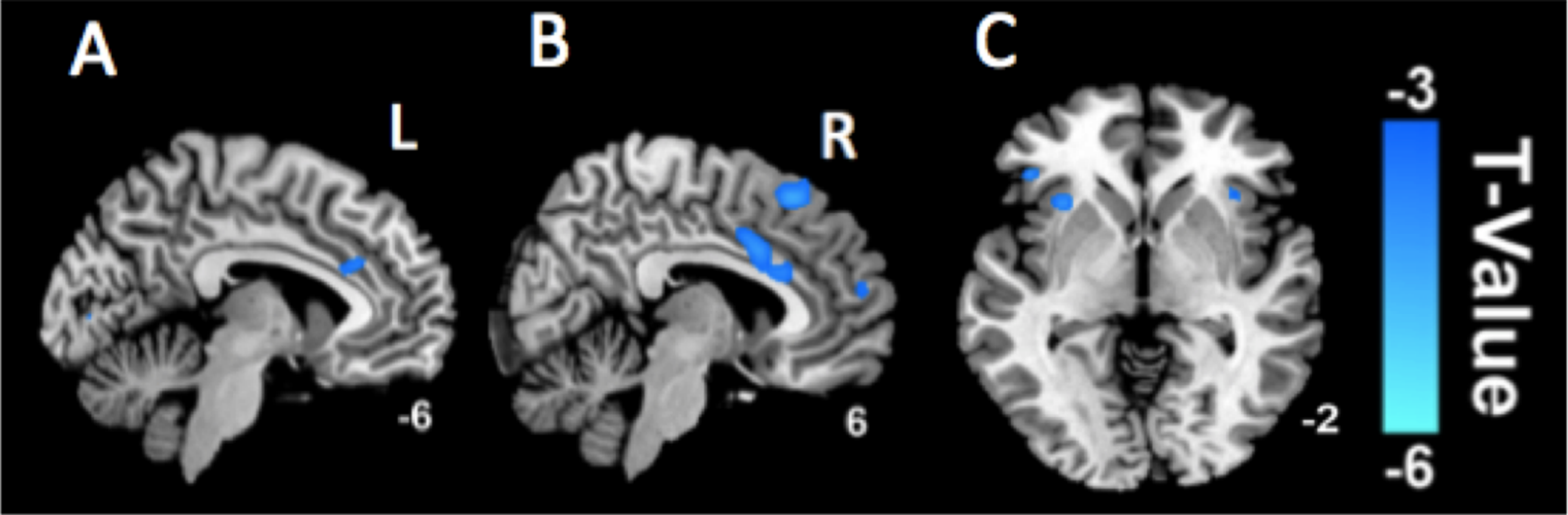
Decreased ReHo values in the alerted brain regions. The CP/CPPS-patients have significantly decreased Reho in the bilateral ACCs (A and B), INCs (C) and right mPFC (B) in comparison with the healthy controls (p < 0.05, false discovery rate corrected).

**Fig 2:**
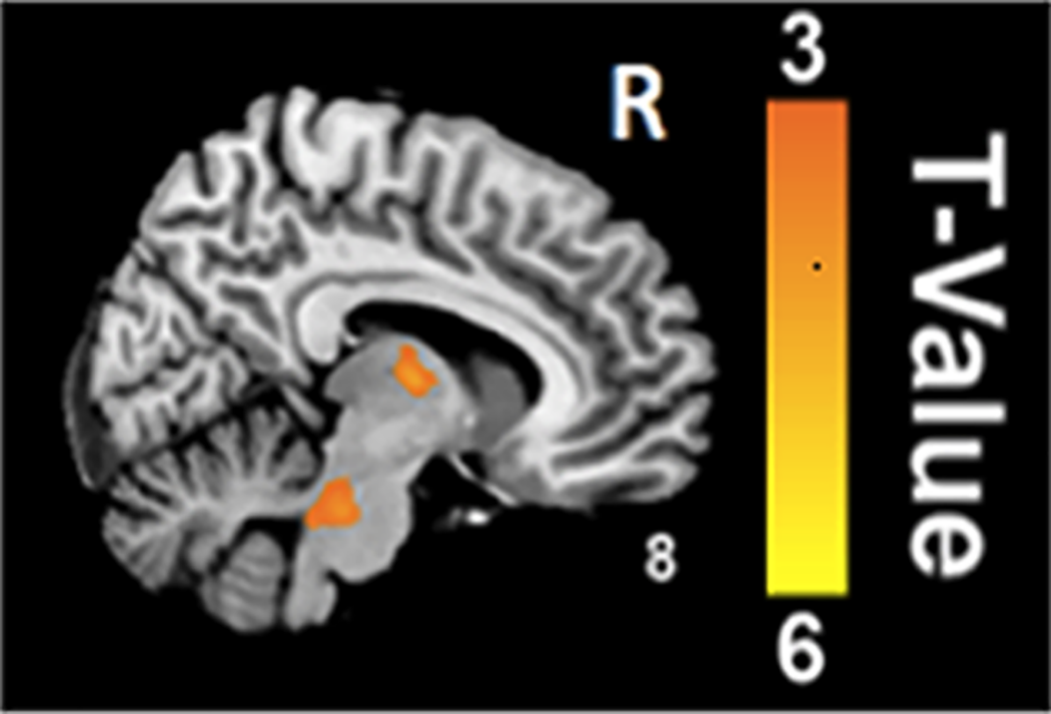
Increased ReHo values in the alerted brain regions. The CP/CPPS-patients have significantly increased ReHo in the brainstem and right thalamus in comparison with the healthy controls (p < 0.05, false discovery rate corrected).

In comparison with the healthy controls, CP/CPPS-patients had significantly decreased Reho in the bilateral ACCs (Fig 1A and 1B), INCs (Fig 1C) and right medial prefrontal cortex (mPFC) (Fig 1B), while significantly increased ReHo in the brainstem and right thalamus (Fig 2) (p < 0.05, false discovery rate corrected).

### Behavioral measurements

Table 1 demonstrated the ReHo values of alerted brain regions in the CP/CPPS-patients compared to the healthy controls. The behavioral measurements were compared between the CP/CPPS-patients and the healthy controls (Table 2). There was no significant difference in the age between the CP/CPPS-patients and the healthy controls (p = 0.98).

**Table 1:** Brain regions showing ReHo differences in the included CP/CPPS-patients compared to the healthy controls.

Brain region	Hemisphere	BA	MNI			T-Value	Voxel
			X	Y	Z		
**Patients > HCs**							
Thalamus	R		9	-9	6	4.36	27
Brainstem	L		-12	-30	-30	4.30	16
	R		6	-30	-27	3.68	25
**Patients < HCs**							
ACC	L	24	-6	27	24	-3.67	74
	R	24	9	15	33	-3.87	123
INC	L	47	-33	18	0	-4.00	32
	R	47	33	24	0	-3.82	30
mPFC	R	10	6	57	15	-3.61	10

ReHo: regional homogeneity; CP/CPPS: Chronic prostatitis/Chronic pelvic pain syndrome; BA: Brodmann area; MNI: brain coordinates from the Montreal Neurological Institute space; HC: healthy controls; ACC: anterior cingulate cortex; INC, insular cortex; mPFC, medial prefrontal cortex.

**Table 2:** Comparisons of behavioral measurements between the included CP/CPPS-patients and healthy controls.

Behavioral measurement	CP/CPPS-patients(Mean ± SD)	Healthy controls (Mean ± SD)	p value
**Age (years)**	34.1 ± 10.3	34.1 ± 10.4	0.98
**Education (years)**	10.7 ± 2.9	10.7± 2.8	0.93
**Duration of CP/CPPS (years)**	4.0 ± 1.6	0	-
**NIH-CPSI total score**	25.7 ± 6.8	0	-
**NIH-CPSI pain subscale**	11.1 ± 4.2	0	-
**SAS standard score**	45.2 ± 13.0	29.7 ± 5.2	< 0.001
**SDS standard score**	49.9 ± 12.9	31.0 ± 6.1	< 0.001

CP/CPPS: Chronic prostatitis/Chronic pelvic pain syndrome; SD: standard deviation; NIH-CPSI: National Institutes of Health Chronic Prostatitis Symptom Index; SAS: Zung Self-Rating Anxiety Scale; SDS: Zung Self-Rating Depression Scale.

### Relationships between mean ReHo values and behavioral measurements

**Fig 3:**
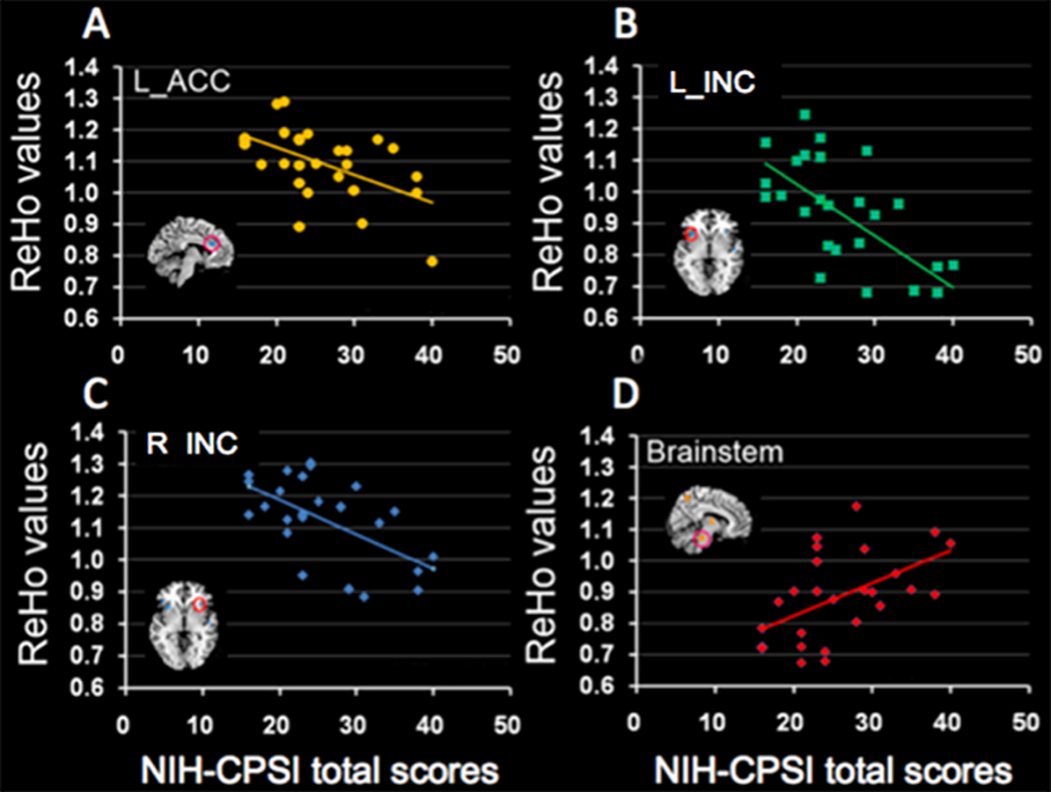
Relationships between mean ReHo values and NIH-CPSI total scores. In the CP/CPPS-patients, there were significantly negative correlations between the NIH-CPSI total score and the mean ReHo value in the left ACC (r = -0.52, p = 0.005) (A), left INC (r = -0.64, p < 0.001) (B) and right INC (r = -0.57, p = 0.002) (C), while a significantly positive correlation between the NIH-CPSI total score and the mean ReHo value in the brainstem (r = 0.52, p = 0.005) (D).

**Fig 4:**
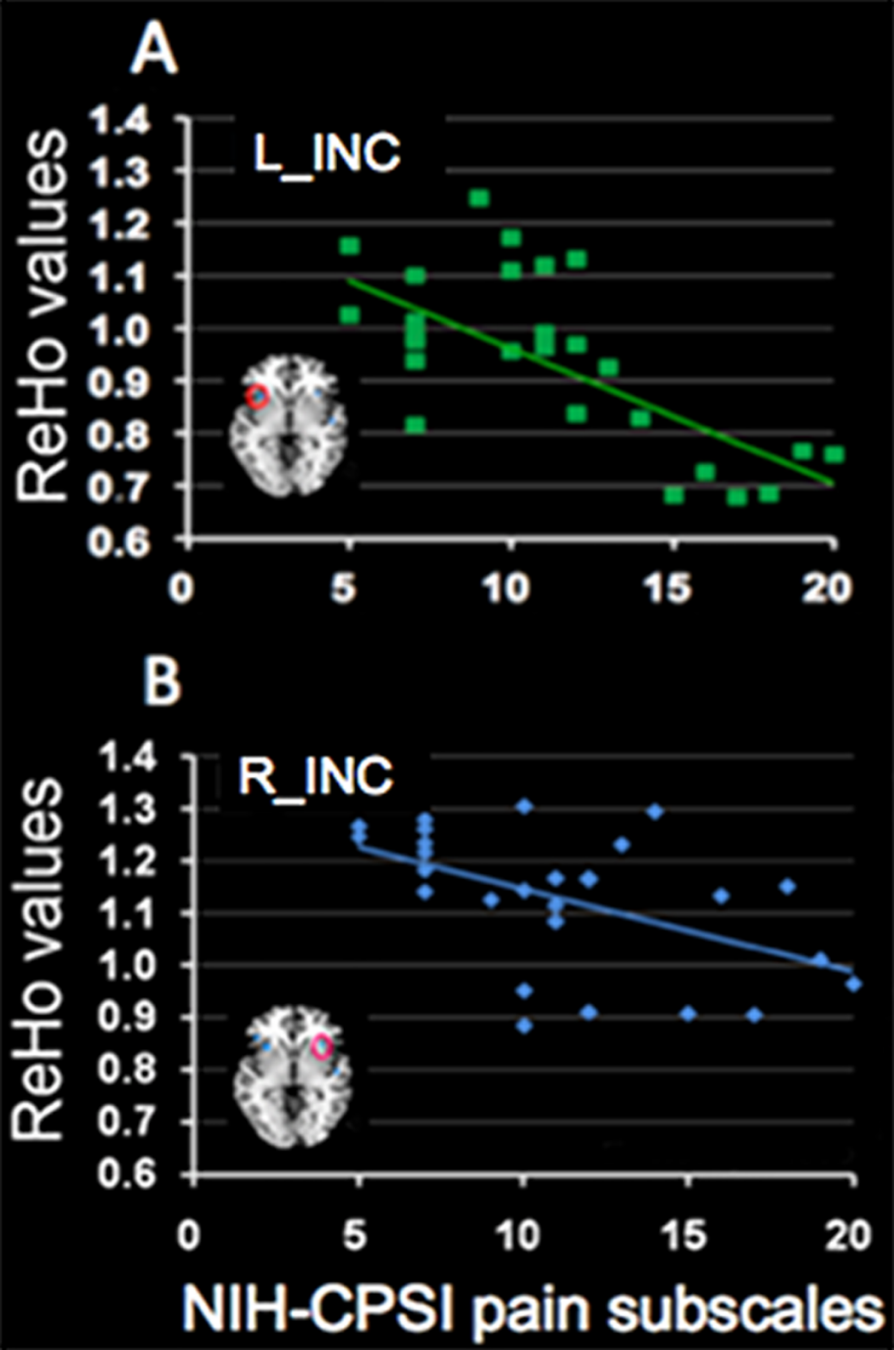
Relationships between mean ReHo values and NIH-CPSI pain subscales. In the CP/CPPS-patients, there were significantly negative correlations between the NIH-CPSI pain subscale and the mean ReHo value in the left INC (r = -0.62, p = 0.001) (A) and right INC (r = -0.51, p = 0.006) (B).

In the CP/CPPS-patients, NIH-CPSI total score had significantly negative correlations with the mean ReHo value in the left ACC (r = -0.52, p = 0.005) (Fig 3A), left INC (r = -0.64, p < 0.001) (Fig 3B) and right INC (r = -0.57, p < 0.002) (Fig 3C), while a significantly positive correlation with the mean ReHo value in the brainstem (r = 0.52, p = 0.005) (Fig 3D). The NIH-CPSI total score had no significantly correlations with the mean ReHo value in the right thalamus (p > 0.05/8). In addition, the NIH-CPSI pain subscale had significantly negative correlations with the mean ReHo value in the left INC (r = -0.62, p = 0.001) (Fig 4A) and right INC (r = -0.51, p = 0.006) (Fig 4B). The NIH-CPSI pain subscale had no significantly correlations with the mean ReHo value in the left ACC, right thalamus and brainstem (all p > 0.05/8).

## Discussion

In the present study, our results showed that the CP/CPPS-patients had significantly decreased ReHo in the bilateral ACCs, INCs and right mPFC, while increased ReHo in the brainstem and right thalamus compared with the healthy controls. These findings may be helpful for further revealing the neural mechanism of CP/CPPS.

A number of previous studies have shown that the ACC [15,16] and INC [17–19] are involved in the pain processing. The ACC and INC were always consistently activated during pain [17–20]. Farmer et al [8] reported that the INC was activated during spontaneous pelvic pain in the CP/CPPS-patients. However, our current results showed that the bilateral ACCs and INCs had decreased ReHo in the CP/CPPS-patients compared with the healthy controls. Our contradictory results may be caused by the patients without spontaneous pelvic pain during MRI scanning. Our results may provide a new insight into the intrinsic neural mechanism of CP/CPPS. ACC and INC are essential for descending pain regulation [15]. They are implicated in the endogenous opioid analgesia system that is a critical neurotransmitter system for modulating pain process [21–23]. In this study, the decreased ReHo of the bilateral ACCs and INCs indicated that the impaired descending pain inhibition existed in CP/CPPS. To support our proposal, the deficient pain inhibitory also existed in some other chronic pain state such as fibromyalgia that had decreased activation in the bilateral ACCs [24]. Both of ACC and INC have high opiate receptor binding potentials to suppress pain [21]. Relief of pain was associated with the increased concentration of opioid receptors availability in the ACC and INC [25]. Therefore, we suggest that the decreased ReHo of the bilateral ACCs and INCs in CP/CPPS predicts the reducing efficacies of endogenous opioid receptors. These results were consistent with the findings from previous studies that the chronic pain diseases had the decreased opioid receptor availability [26,27]. The reduced opioid binding was most likely dominated by the decreased number and density of available opioid receptors [28]. Persistent analgesic activation for increased release of endogenous opioids in the bilateral ACCs and INCs may ultimately lead to an inefficient opioid analgesia system in CP/CPPS. Gamma-aminobutyric acid is another kind of inhibitory neurotransmitter, which could be reduced by chronic pain in the INC [29]. The decreased level of gamma-aminobutyric acid within the INC may further worsen the impaired descending pain inhibition.

In addition, we found that the mean ReHo value of left ACC and bilateral INCs was inversely correlated with the NIH-CPSI total score. Therefore, we suggest that the impaired descending pain inhibition lead to insufficiently suppressing nociceptive input in CP/CPPS. Moreover, the left ACC in the dominant hemisphere may have a closer relationship with the regulation than the right ACC in CP/CPPS. It was consistent with the results of a previous study reported by Mordasini et al [9] that the relative gray matter volume of left ACC was decreased in the CPPS-patients and correlated with the illness bother. The ACC could modulate both of pain intensity and pain unpleasantness [30].

The mPFC, especially in the right hemisphere, is also involved in the descending pain regulation [31]. The activity of the right prefrontal cortex was related to analgesia, leading to decreased pain perception [31]. Our results showed that the right mPFC had decreased ReHo in the CP/CPPS-patients compared with the healthy controls. It indicated that the decreased ReHo in the right mPFC led to insufficient analgesia. In addition, the gamma-aminobutyric acid dysfunction of mPFC induces abnormal gamma oscillations, which may affect the perception of pain [32]. Furthermore, the mPFC had increased connectivity with ACC and INC in the chronic pain [33].

The ascending pain pathways also play important roles in the pain modulation [25]. There exists an ascending pain pathway which projects from spinal cord to brainstem as far as thalamus [34]. Brainstem is important for receiving nociceptive signals that ascend from spinal cord. Thalamus is the gateway for transmitting the nociceptive information to the cerebral cortices [19,35,36]. The nociceptive information flows reach ACC and other cortical regions through thalamus [37]. In addition, Labus et al [38] reported that the right thalamus was more critical for controlling information flows in the patients with irritable bowel syndrome than that in the healthy controls. In this study, the increased ReHo in the brainstem and right thalamus of the CP/CPPS-patients could contribute to the hyperalgesia. Our findings were consistent with the results of previous study that the patients with chronic low back pain had lower pain threshold compared to the healthy controls [39]. The nociceptive information may be exaggerated and transmitted through the brainstem and right thalamus to the cerebral cortices in CP/CPPS. Hence, the perception of pain is sensitive in the CP/CPPS-patients because of augmented nociceptive signals.

Brainstem is also important in the descending pain regulation that could be disrupted by the chronic pain [40]. In this study, the increased ReHo of brainstem indicated that the ascending pain hyperalgesia was predominant compared to the descending pain inhibition in CP/CPPS [41]. The imbalance between the descending and ascending pain pathway in the brainstem may facilitate the pain perception in CP/CPPS.

This study had some limitations. First, we only used the volume-based ReHo method to examine the neural mechanism of CP/CPPS in the current study. However, the partial volume effects were salient in the voxels close to the boundary between the gray matter and white matter [42]. The surface-based ReHo method could address this issue because it was more specific to the intrinsic functional organization of the cortical mantle [42]. Moreover, the surface-based ReHo method had the higher test-retest reliability compared with the volume-based ReHo method [42,43]. In the future, we will further analyze the fMRI data by using the surface-based ReHo method to reflect the functional organization of the cortex more naturally and reduce the intersubject variability [44,45]. Second, we only explored the neural mechanism of CP/CPPS-patients without spontaneous pelvic pain during MRI scanning, the discrepancy of cerebral activity between the state without and with pain in the same population with CP/CPPS should be further researched.

## Conclusions

In conclusion, an impaired pain modulatory system, either by decreased descending pain inhibition or enhanced pain facilitation, may explain the pain symptoms in CP/CPPS.
